# Consequence of aging at Au/HTM/perovskite interface in triple cation 3D and 2D/3D hybrid perovskite solar cells

**DOI:** 10.1038/s41598-020-79659-3

**Published:** 2021-01-08

**Authors:** Zubair Ahmad, Arti Mishra, Sumayya M. Abdulrahim, D. Taguchi, Paek Sanghyun, Fakhra Aziz, M. Iwamoto, T. Manaka, Jolly Bhadra, Noora J. Al-Thani, Mohammad Khaja Nazeeruddin, Farid Touati, Abdelhak Belaidi, Shaheen A. Al-Muhtaseb

**Affiliations:** 1 Center for Advanced Materials (CAM), Qatar University, 2713 Doha, Qatar; 2 Department of Electrical and Electronic Engineering, Tokyo Institute of Technology, 2-12-1 O-okayama, Meguro-ku, Tokyo, 152-8552 Japan; 3 Department of Chemistry and Energy Engineering, Sangmyung University, Seoul, 03016 Republic of Korea; 4 Jinnah College for Women, University of Peshawar, Peshawar, 25120 KPK Pakistan; 5 Qatar University Young Scientists Center (YSC), Qatar University, 2713 Doha, Qatar; 6 Group for Molecular Engineering of Functional Materials, Institute of Chemical Sciences and Engineering, EPFL VALAIS, 1951 Sion, Switzerland; 7 Department of Electrical Engineering, College of Engineering, Qatar University, 2713 Doha, Qatar; 8 Department of Petroleum Engineering, Texas A&M University at Qatar, Education City, 23874 Doha, Qatar; 9 Department of Chemical Engineering, College of Engineering, Qatar University, 2713 Doha, Qatar

**Keywords:** Energy science and technology, Engineering, Materials science, Physics

## Abstract

Perovskite solar cells (PSCs) expressed great potentials for offering a feasible alternative to conventional photovoltaic technologies. 2D/3D hybrid PSCs, where a 2D capping layer is used over the 3D film to avoid the instability issues associated with perovskite film, have been reported with improved stabilities and high power conversion efficiencies (PCE). However, the profound analysis of the PSCs with prolonged operational lifetime still needs to be described further. Heading towards efficient and long-life PSCs, in-depth insight into the complicated degradation processes and charge dynamics occurring at PSCs' interfaces is vital. In particular, the Au/HTM/perovskite interface got a substantial consideration due to the quest for better charge transfer; and this interface is debatably the trickiest to explain and analyze. In this study, multiple characterization techniques were put together to understand thoroughly the processes that occur at the Au/HTM/perovskite interface. Inquest analysis using current–voltage (I–V), electric field induced second harmonic generation (EFISHG), and impedance spectroscopy (IS) was performed. These techniques showed that the degradation at the Au/HTM/perovskite interface significantly contribute to the increase of charge accumulation and change in impedance value of the PSCs, hence resulting in efficiency fading. The 3D and 2D/3D hybrid cells, with PCEs of 18.87% and 20.21%, respectively, were used in this study, and the analysis was performed over the aging time of 5000 h. Our findings propose that the Au/HTM/perovskite interface engineering is exclusively essential for attaining a reliable performance of the PSCs and provides a new perspective towards the stability enhancement for the perovskite-based future emerging photovoltaic technology.

## Introduction

Three-dimensional (3D) hybrid perovskite materials have rapidly emerged as highly promising materials for optoelectronic applications as showcased by photovoltaic technologies. By compositional engineering and fine optimization of the film morphology, PSCs based on 3D mixed cation and anion materials have reached power conversion efficiencies (PCEs) exceeding 25%^1^, launching this technology in the first line along with commercial thin-film semiconductor solar cells. However, overcoming its poor stability (mainly due to the 3D materials' degradation when exposed to moisture, oxygen, heat, and UV radiation) is a critical challenge; hence, it is a major bottleneck for their market application^2^. Several studies have been conducted to address the moisture sensitivity issue associated with 3D perovskites. There have been attempts to modify the 3D perovskite layer by tuning its chemical composition^3–6^. Moreover, there have been attempts to alter the interface between HTM and the perovskite layer^7,8^. This restricts electron/hole recombination and, at the same time, resists moisture, increasing the stability of PSCs. Some studies are dedicated to passivating PSCs by polymers. Hwang et al*.*^9^ employed polytetrafluoroethylene, which is a hydrophobic polymer, and spin-coated it on top of the perovskite layer to act as a passivation agent towards moisture. Similarly, Habisreutinger et al.^10^ employed a combination of poly(methyl methacrylate) and carbon nanotubes as a hole transporting material (HTM). By analyzing the degradation processes with time, the results proved an increased resistance to moisture. These studies demonstrated moderate stabilities, but at the expense of low PCEs. Since the HTM is known to be one of the significant sources of degradation in PSCs, HTM-free PSCs have also been consistently proposed in previous studies^8,11–14^; however, the PCEs of this type of PSCs are still low (< 15%).

A critical role of the metal/HTM/perovskite interface in the n-i-p structured PSCs has also been proven, and the stability and PCE could be improved by altering or modifying the interface between the HTM and perovskite. An interface layer between HTM/perovskite can successfully prevent the chemical or mechanical degradation of the interface. The 2D perovskite layers have been in the spotlight (as capping layers) due to their remarkable moisture resistance property. Indeed, employing it as a passivating layer over the 3D perovskite emerged as a potential source of stability enhancement in PSCs with a moderate device performance^2,15–19^. The 2D perovskite layer is usually prepared by inserting a large-sized hydrophobic cation spacer into the perovskite crystal lattice, which can effectively hinder the moisture intrusion and improve stability of the PSCs. Many groups investigated the optimization of 2D/3D hybrid perovskite structures. Grancini et al.^16^ engineered a 2D/3D composite by mixing and infiltrating the precursors by a single step deposition and reported a PCE of 14.6% with one-year stability. Subsequently, Wang et al.^18^ developed a 2D/3D butylammonium-cesium-formamidinium lead halide perovskite by testing different precursor compositions and achieved a PCE of 17.3% with a 1000 h stability in air. Directly mixing bulky organic cations with the 3D perovskite precursor has not been proven to enhance the PSCs' performance. This is because the charge transport efficiency decreases significantly due to the quantum confinement effect^2^. Other groups have chosen a layer by layer deposition technique to deposit the 2D perovskite layer on top of the 3D perovskite layer. Chen et al*.*^2^ fabricated a 2D/3D perovskite layered structure by in situ growth of 2D capping layers on the 3D perovskite film. The PSCs were analyzed after 1000 h in an ambient environment; were found to retain almost 90% of their initial power conversion efficiencies. Cho et al.^19^ also fabricated a layered structure by employing a 2D perovskite layer with a wide band-gap on top of the 3D perovskite layer and demonstrated a PCE 20.1%. It retained almost 85% of this PCE after exposure to 1 sun illumination for 800 h. Ahmad et al.^15^ compared the stability of 3D PSCs with the layer by layer 2D/3D hybrid PSCs by electric field induced second harmonic generation (EFISHG) and impedance spectroscopy (IS) techniques under light and heat soaking conditions. Their results also show that a layer-by-layer 2D/3D hybrid structure is more stable than the 3D perovskite cells.

The capping of the 3D perovskite by the 2D layer improves stability. It prevents the perovskite layer from degrading (and thus slow down the PSCs' degradation rate) due to its moisture resistance property. Still, the question remains: why the 2D/3D hybrid cell degrades gradually at room temperature and even in dark conditions. Therefore, this study is dedicated to getting insights into the layer's interfaces deposited over the 2D perovskite layer (Au/HTM/perovskite). The present work reveals the reasons restricting the stability at Au/HTM/perovskite interface in the 3D and 2D/3D hybrid PSCs. We use multiple non-destructive approaches (including the I-V, EFISHG, and IS measurements) to elucidate the factors limiting PSCs' performance over the 5000 h period. Although many studies used the I-V and IS techniques individually or together^19,20^ for the analysis of 3D and 2D/3D PSCs, the primary incentive of our work is the lack of previous studies on the comparison of 3D and 2D/3D hybrid PSCs in terms of their complicated charge dynamics processes at the Au/HTM/perovskite interface over time. Our findings elucidate that the degradation due to the chemical instability of the Au/HTM/perovskite is a fundamental reason behind the steady efficiency fading of the n-i-p structured PSCs. Our understanding obtained from the present study can provide the direction for a more critical assessment of PSCs stability.

## Experimental procedure

### Fabrication of PSCs

Both the 3D and 2D/3D hybrid PSCs were fabricated on FTO coated glass substrates (Nippon sheet glass). The electron transport layer (c-TiO_2_) was deposited by Spray pyrolysis using titanium diisopropoxide (Sigma-Aldrich) precursor in 2-proponal (1:10, v/v) at 450 °C. A100 nm thick meso-TiO_2_ (m-TiO_2_) layer was coated using spin-coating of TiO_2_ paste (Dyesol 30 NR-D) diluted in ethanol (1:8, w/v) followed by annealing at 500 °C for 30 min. The tin oxide passivation layer was deposited using a solution of 1.2% SnCl_4_ (Acros) in water and then baked at 180 °C for 60 min. The UV-ozone treatment was performed prior to each c-TiO_2_, m-TiO_2_, and perovskite layer. Triple cation perovskite precursor was prepared using 1.15 M PbI_2_ (TCI), 1.1 M FAI (Dyesol), 0.2 M MABr (Dyesol) 0.2 M PbBr_2_ (TCI) and 1.15 M CsI (GmbH) in DMF and DMSO (4:1,v/v) mixed solvent.

**Figure 1:**
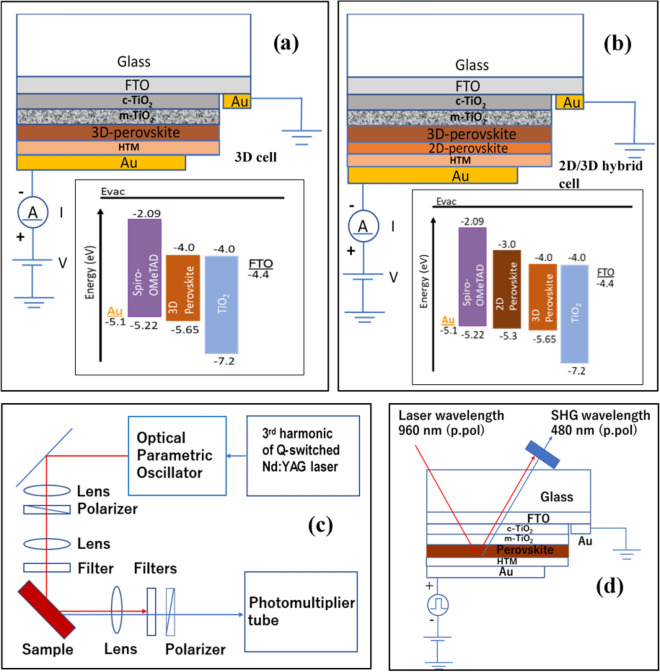
Device diagrams and schematic of the connection during the measurements. **(a)** 3D samples and **(b)** 2D/3D hybrid samples. The insets show the energy band-gap diagram of perovskite solar cells (PSCs). **(c)** shows the schematic of the EFISHG measurement system used to analyze both types of the samples, and **(d)** gives the illustration of the orientation of the 3D samples (a similar setup was used for the 2D/3D hybrid samples.

A 37 µl of the prepared precursor solution was spin-coated on an m-TiO_2_ layer (in a glove box) using a two-step spinning procedure: first at 2000 rpm for 10 s, then at 5500 rpm for 30 s. Chlorobenzene (110 µl) was used as an antisolvent after finishing 25% of 2^nd^ phase of spinning. Crystals of 3D perovskite were developed after 90 min of annealing at 100 °C. In the case of 2D/3D hybrid cells, the 2D perovskite layer was spun-cast over the 3D perovskite layer at the speed of 4200 rpm for 20 s using the 1.5 M of PEAI in isopropanol. The spiro-OMeTAD (as hole transport layer (HTL)) was deposited over the perovskite absorber layer at a spinning speed of 4000 rpm for 30 s. The HTL precursor was prepared using 78.2 mg spiro-OMeTAD in 1 mL chlorobenzene, 0.03 M Co[*t*-BuPyPz]_3_[TFSI]_3_ (FK209) in acetonitrile, 0.5 M Li-TFSI in acetonitrile and 31.28 µL of 4-tert-butylpyridine. The device fabrication was completed by depositing the 70 nm thick Au electrode by the thermal evaporation method. Figure 1a, b show the schematic of the fabricated 3D and 2D/3D hybrid devices, respectively, while the insets show the respective energy level diagrams. The energy level diagrams of the 3D and 2D/3D samples (given in Fig. 1) show that charge transport resistance provided by the HTM/perovskite interface must be small in 2D/3D samples because the 2D layer offers an extra step to the charge carriers.

### PSCs characterization

The current–voltage (*I–V*) curve of the 3D and 2D/3D PSCs were obtained by measuring current in the response of an externally applied biasing voltage by a digital Source Measurement Unit (Keithley 2400) at the scan rate of 25 mV/s in the forward (from 0 to + V) direction. A 25 mV/s forward and reverse voltage scan rate has been used. The PSCs have not been preconditioned (e.g., by light and heat-absorbing or being kept on long forward biasing) before measurement. A solar illumination simulator (Oriel, 450 W Xenon, AAA class) was used during the *measurement of the J–V characteristics*. Light intensity was calibrated using a Si reference cell that contained an IR-cut-off filter (KG5, Newport) and was fixed at one sun. To reduce the scattering of light, the PSCs have been masked with a 0.16 cm^2^ active area. The impedance spectroscopy (IS) measurements (in the frequency range from 1 to 0.1 MHz) were performed with Gamry-3000 potentiostat. A sinusoidal AC potential perturbation of 10 mV was overlaid over the applied DC bias potential under dark and light conditions. During the measurements, the devices were kept in the dark faradaic cage to ensure electrical isolation; and the measurements were repeated for three cycles during the 5000 h. The resultant impedance spectra have been fitted with Gamry Echem Analyst and ZMAN software. The EFISHG technique was used to study the charge accumulation at the interfaces, underlying device physics of the PSCs. The experimental setup of the EFISHG measurement system mainly consists of a Q-switched Nd: YAG laser attached with an optical parametric oscillator (OPO) and a photomultiplier tube, as shown in Fig. 1c. A rectangular AC square voltage pulse (as shown in Fig. 1d) that consists of a 100 µs pulse width was applied to the Au/HTM electrode. Here it is important to note that the IS represents the ac response, while EFISHG detects the electrostatic d.c. response.

## Results and discussion

**Figure 2:**
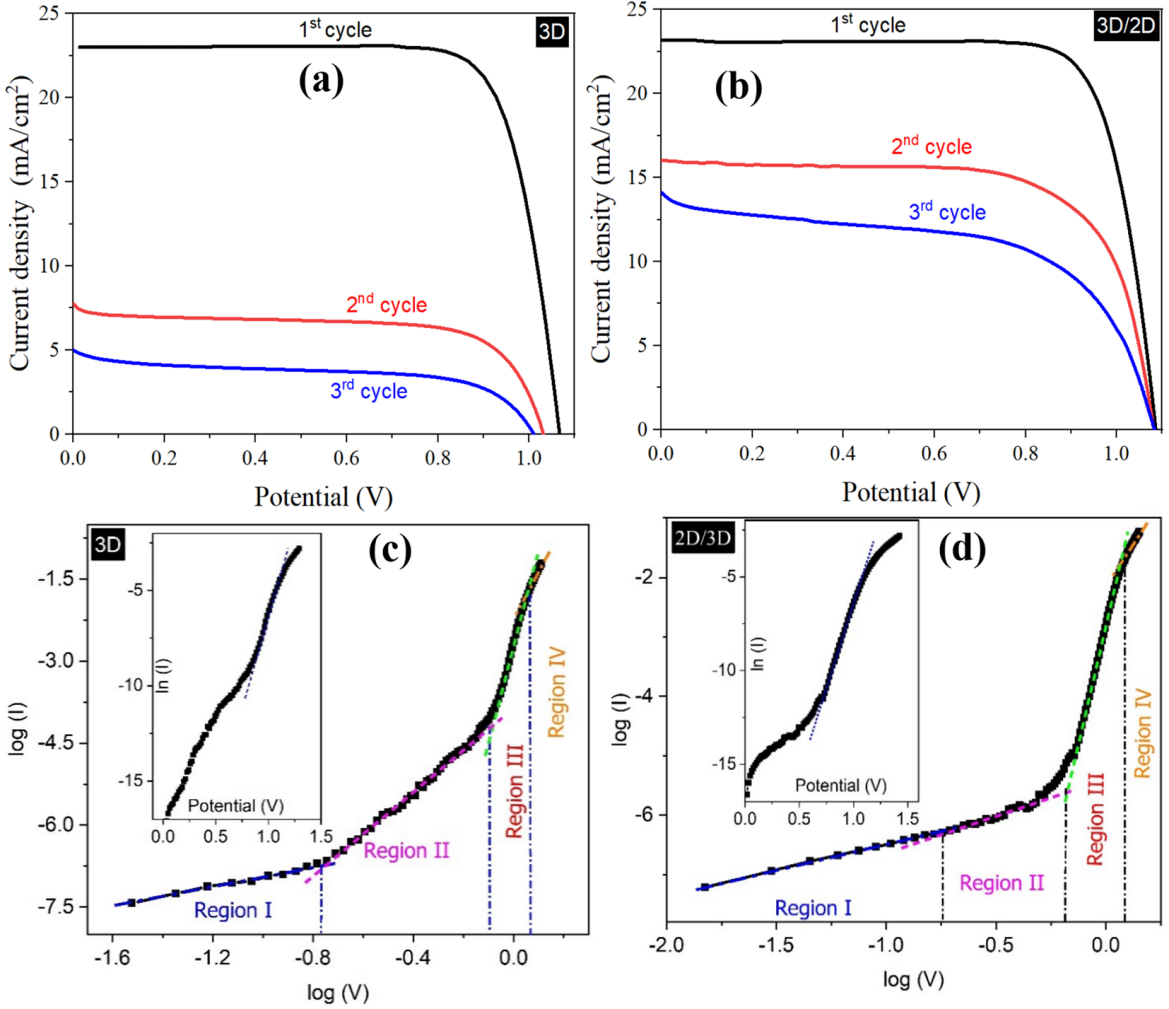
**(a,b)** J–V curves of 3D and 2D/3D perovskite solar cells under 1 Sun illumination (100 mW/cm^2^), respectively. The J–V curves were recorded in three cycles at different intervals of time (fresh, 2500 h & 5000 h) to compare the change in the photovoltaic parameters of both 3D and 2D/3D samples. The J–V characteristics were recorded in the forward (from 0 to + V) direction at a scan rate of 25 mV/s. **(c,d)** represents the I–V curves of 3D and 2D/3D perovskite solar cells, respectively, under dark conditions.

Figure 2a, b shows the J–V characterization of the 3D and 2D/3D samples, respectively. The J–V characteristics were recorded over the different intervals of time, including fresh samples (1st cycle), after 2500 h (2nd cycle), and after 5000 h (3rd cycle) of fabrication. The fresh 3D PSCs exhibited a short circuit current density (J_sc_) of 23.18 mA/cm^2^, an open-circuit voltage (V_oc_) of 1.07 V, and a fill factor (FF) of 77%. This yielded a power conversion efficiency (PCE) of 19.01%. However, after keeping the samples in the indoor ambient conditions for 2500 h, the PCE decreased to 5.16%; and after 5000 h, the PCE further reduced to 2.70%. Overall, in the case of 3D samples, the J_sc_ dropped from 23.18 to 4.99 mA/cm^2^, V_oc_ decreased from 1.07 to 1.025 V, and FF changed from 78.3 to 53.4% within 5000 h. On the other hand, the J–V characterization analysis of fresh 2D/3D PSCs, J_sc_, V_oc_, FF, and PCE values were found to be 23.29 mA/cm^2^ 1.08 V, 79.2%, and 19.8%, respectively. After the first 2500 h, the PCE of the 2D/3D hybrid samples decreased to 12.04%, then to 8.60% in the next 2500 h while the value of their V_oc_ did not decrease as opposed to the V_oc_ of 3D PSCs; and the reduction in the PCE of 2D/3D PSCs was much slower as compared to the 3D PSCs. This was expected as the 2D perovskite acts as a passivating layer on top of the 3D perovskite layer. This prevents the 3D perovskite from degrading by restricting moisture penetration, allowing them to stay stable over long periods^2^. Table 1 shows the change in the photovoltaic parameters of both 3D and 2D/3D PSCs over time.

**Table 1:** Photovoltaic parameters of the 3D and 2D/3D samples extracted for the three cycles within the period of 5000 h with the difference of 2500 h.

Samples		J_sc_ (mA/cm^2^)	V_oc_ (V)	FF (%)	PCE (%)
3D	1st cycle	23.2 ± 0.2	1.07 ± 0.015	78.3 ± 0.4	19.2 ± 0.4
	2nd cycle	7.77 ± 0.23	1.03 ± 0.017	64.4 ± 0.45	5.2 ± 0.5
	3rd cycle	4.99 ± 0.30	1.02 ± 0.022	53.4 ± 0.50	2.7 ± 0.72
2D/3D	1st cycle	23.3 ± 0.2	1.085 ± 0.01	79.2 ± 0.30	19.8 ± 0.3
	2nd cycle	16 ± 0.22	1.08 ± 0.012	69.7 ± 0.33	12 ± 0.45
	3rd cycle	14.1 ± 0.25	1.08 ± 0.014	56.4 ± 0.35	8.6 ± 0.55

In the 3D sample, the value of the V_oc_ dropped from 1.07 to 1.02 V within 5000 h. Using the Maxwell–Wagner (MW) effect model^21,22^, the relation between the accumulated charges and V_oc_ can be discussed. Once the photogenerated excitons dissociate into free electrons and holes, the holes spread in the HTM layer with a time of "t_rh_" and the electrons spread in the electron transport layer (ETL) with a time of "t_re_" and thus produce the voltage V_oc_. Then, excess charge carriers accumulate at the interfaces due to the difference in dielectric relaxation times of the holes and electrons, which express their spreading times in a fashion as Q_s_ = J_sc_(t_rh_ − t_re_), (positive current J_sc_ is defined as that flows from FTO to Au in the solar cell). This phenomenon is called the MW effect, which describes the charge accumulation at the two different materials' interface. In other words, excess carriers accumulate at the interface when the carrier spreading times in the two adjacent materials are different. The electrons accumulate if the (${t}_{\mathrm{rh}}<{t}_{\mathrm{re}}$ and ${J}_{\mathrm{sc}}>0$), and holes accumulate when (${t}_{\mathrm{rh}}>{t}_{\mathrm{re}}\mathrm{ and }{J}_{\mathrm{sc}}>0)$). Under illumination, the electron–hole pairs are disassociated at the TiO_2_/perovskite interface. The disassociated holes and electrons go towards the Au and FTO electrodes, respectively, and generate a V_oc_ > 0 at the Au electrode in reference to the grounded FTO electrode. We assume that, in the case of 3D samples, the excess holes are accumulated at the perovskite/HTM interface as obvious from the decrease in the V_oc_ with time. The accumulation of excess holes forms an electrostatic potential at the molecular interface and leads to a decrease in the V_oc_ in the 3D samples. In the 3D samples, holes ${Q}_{s}=$ 4.1 × 10^–9^ C/cm^2^ accumulate at the layers interface with ${J}_{sc}=$ + 23.2 × 10^–3^ A/cm^2^. As a result, charge-separated electrons additionally lose electrostatic energy $\mathrm{e}\Delta V=\mathrm{e}{Q}_{s}/({C}_{1}+{C}_{2})$ to move to FTO electrode ($\Delta V$: voltage loss, ${C}_{1}$ and ${C}_{2}$: capacitance of the PSCs in the 1st and 3rd cycle, respectively). This electrostatic energy loss is calculated as $e\Delta V=$ 0.051 ± 0.001 eV with values ${C}_{1}={C}_{2}=$ 3.99 ± 0.1 × 10^–8^ F/cm^2^. On the other hand, in 2D/3D samples, the electrostatic energy loss is small, i.e., $e\Delta V=0.0052\pm 0.0002$ eV (${Q}_{s}=$ 0.68 × 10^–9^ C/cm^2^, ${C}_{1}={C}_{2}=$ 6.57 ± 0.03 × 10^–8^ F/cm^2^). Consequently, the V_oc_ of 2D/3D samples remains ~ 0.05 V higher than that of 3D. The 2D layer prevents the charge accumulation at the perovskite/HTM interface, and hence the V_oc_ almost remains constant.

To analyse the charge conduction probabilities in the 3D and 2D/3D PSCs, the J-V characteristics (in the dark) are presented in the double-logarithmic and semi-logarithmic scales, as shown in Fig. 2c, d. In the case of both 3D and 2D/3D samples, the four distinct regions can be observed, and the current is governed by the power-law (I ∝ V^m^), where "m" represents the slope of the curve in the double-logarithmic scale of the J–V characteristics. In 3D samples, at low voltages, from 0 to 0.15 V, the current increases linearly (slope = 1) with an increase in both samples' bias voltage. This region represents the ohmic conduction phenomenon due to the drift of the thermally generated free charge carriers. In the voltage range between 0.15 and 0.75 V (region II), the current is proportional to V^3.8^. This 2nd region of the curves can be assigned to the conduction due to the shallow traps. With a further increase of applied potential, from 0.9 V to 1.1 V (Region III), the current represents the deep traps region in both cycles with the slopes of 17.32 ± 0.22. The voltage range between Region II and Region III represents the transition region between the two conduction phenomena and can be denoted by V_T_. Region IV can be represented by the relationship I ∝ V^9.23^. This means that the traps have started to be filled over the applied potentials of the 1.1 V. This potential range is close to the V_oc_ of the PSCs; hence this indicates a relation between the trapped filled region and V_oc_ of the cell. Here, it is important to mention that in the multi-layer's devices such as perovskite solar cell devices, it is difficult to distinguish whether these traps exist in the perovskite band-gap layer (or in any other layer) or at the interfaces of different layers. In these regions, the charge carriers are injected into the semiconductor by the thermionic process that takes place within the potential barriers at different positions and energetic distribution in the devices. Hence, the total free charge carriers are much less than the entrapped charge carriers, and thus the current shows an exponential increment with the elevated applied potential. Fowler–Nordheim (F–N) tunneling may take place in addition to the thermionic emission in the trapped charge limiting current (TCLC) region. With further high voltages, the trap-filled region can be observed, and the conduction in these devices can be represented by the space charge limiting current (SCLC) model, proposed in the book by Lampert and Mark^23^. The SCLC region can be observed when the applied potential is greater than the average energy associated with traps density. Typically, the intermediate regions between the ohmic and SCLC regions are labelled as the trapped space charge regions, and the TCLC model governs the current. The charge conduction in the TCLC region is dominated by the trapping and de-trapping of charge carriers at both positional and energetic distribution. Here, traps are assumed to be accumulated charges, structural (interstitial) defects and/or impurities, which offer localized states between the PSCs' energy band-gap. These trap states catch the free charges and avert them from taking part in the charge conduction. Though, based on the presented experimental results, it is difficult to get any conclusion. However, the charge is directed from the Au toward the FTO; hence, it can be supposed that the first region represents the conduction at the Au/HTM interface while the 2nd region may represent the HTM/perovskite interface. The 3rd region may portray the existence of the traps in the band-gap of the perovskite layer. However, in the 4th region (close to the V_oc_), the energy band gap of mesoporous TiO_2_ and perovskite decreases, and hence the charge transfer resistance is caused by a cumulative response of all interfaces in the PSCs.

Similar behavior of the double-logarithmic scale graph has been observed in the 2D/3D samples. However, the slopes' values are smaller than those of the 3D samples, as shown in Table 2. This indicated that the addition of the intermediate 2D layer between the HTM and 3D perovskite had reduced this value (see the slope of region II in Table 2). Hence, there is a significant contribution of the traps (resistance provided to the charge transfer) from this interface. By improving this interface, the device quality can be improved. The ideality factor 'n', which is used to evaluate the quality of the devices, has been also given in Table 2. The value of "n" was calculated using the slope of the linear region in the Semi-logarithmic scale graphs shown in the insets of Fig. 2c, d. In the case of 2D/3D samples, the value of "n" is smaller, which reveals that the PSCs possesses better diodic properties; however, the difference between the "n" values for the both 3D and 2D/3D is not too much. In the case of ideal diodes, the value of "n" should be close to "1"^24^.

**Table 2:** The slopes obtained from the double-logarithmic scale graphs of the 3D and 2D/3D samples under dark.

Samples	Slope				Ideality factor (n)
	Region I	Region II	Region III	Region IV	
3D	1.0 ± 0.03	3.86 ± 0.30	17.32 ± 0.22	9.23 ± 0.12	2.35 ± 0.19
2D/3D	0.89 ± 0.08	0.94 ± 0.04	15.01 ± 0.20	6.14 ± 0.12	2.13 ± 0.14

The value of the slope represents the conduction mechanism that happened at the different applied potential within the V_oc_ range. The ideality factor "n" represents the diodic quality of the devices.

It is important to mention here that the I–V characteristic, MW model analysis, charge conduction mechanism, and the role of the different interfaces described above are macroscopic approaches and cannot directly probe accumulated charges and space charge fields formed by accumulated charges. Therefore, it is required to perform further analysis microscopically. Hence, we present the analysis using state-of-the-art techniques, including the EFISH and IS, as described in the following sections.

**Figure 3:**
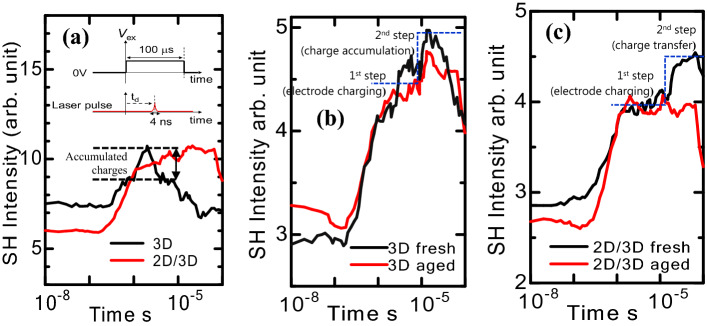
Time-resolved EFISHG results of both 3D and 2D/3D hybrid PSCs. **(a)** shows the 3D and 2D/3D hybrid samples; the dotted lines' difference presents the charge accumulation in the 3D sample. **(b,c)** illustrates the time-resolved EFISHG measurements of the fresh and aged 3D and 2D/3D hybrid samples. AC square wave (− 3 V) with a pulse width of 100 µs was applied to the Au electrode with reference to FTO.

The EFISHG is a well-recognized method to evaluate the excess charge accumulating at the interfaces of the solar cells^25,26^. This technique is adept at probing the electrostatic effect of M-W style interfaces of the PSCs^27^. Using the EFISHG technique, we determined the transit time of charge accumulation (movement) at the Au/HTM/perovskite interface. Figure 3 shows the EFISHG results (time-resolved) of both 3D and 2D/3D hybrid PSCs using the 100 µs voltage pulse width of the applied square AC wave signal. An incident laser light beam with a wavelength of 960 nm was used to probe the electrostatic electric fields in both types of samples. Under the applied square AC wave, the charge $\pm {Q}_{e}$ induced on Au electrode and mesoporous TiO_2_ electrode, respectively, and thus, an electrostatic electric field ${E}_{0}={Q}_{e}/\varepsilon$ was produced in the PSCs, where $\varepsilon$ represents the dielectric constant of the perovskite material. Here, it is important to note that ${E}_{0}>0$ means that the electric field is steering from the Au electrode to the FTO side. Subsequently, charges are infused from the Au surface to the HTM layer, and these charges ${(Q}_{s})$ accumulate at the HTM/perovskite interface and produce an electrostatic electric field of $(1/{d}_{2}){(Q}_{s}/({C}_{1}+{C}_{2}))$ where ${d}_{2}$ represents the perovskite layer thickness, while the ${C}_{1}$ and ${C}_{2}$ represents the capacitances of HTM and perovskite layers, respectively. Hence, the total electric field in the PSCs change to ${{E}^{^{\prime}}}_{0}=({Q}_{e}/\varepsilon )+(1/{d}_{2}){(Q}_{s}/({C}_{1}+{C}_{2}))$. During the EFISHG experiments, we probe the electric field ${E}_{0}$(${E{^{\prime}}}_{0}$) because the intensity of the 2^nd^ harmonic wave is directly promotional to both ${E}_{0}$_,_ (${I}_{shg}\propto {\left|{E}_{0}\right|}^{2}$) and ${E{^{\prime}}}_{0}$_,_ (${I}_{shg}\propto {\left|{E{^{\prime}}}_{0}\right|}^{2}$), whereas the ${E}_{0}$ and ${E{^{\prime}}}_{0}$ originate directly from the electrode charging (${Q}_{e}$) and accumulated charges ${Q}_{s}$, correspondingly.

When the external electric current was applied, the intensity of the SHG signal was noticed to rise promptly after 10^–6^ s (10^–6^ s was the time constant of the measurement circuit). The electric field is developed in the perovskite film due to the electrode charge ${Q}_{e}$, and as soon as the charge carriers move into the HTM layer, these infused charge carriers ${Q}_{s}$ build a space-charge electric field at the HTM/perovskite interface and change the resultant electric field. In the case of 3D samples, when the resultant field is changed, the intensity of the SHG intensity starts to reduce immediately. Explicitly, the results showed that the intensity of the SHG signals rose up to 10^–6^ s due to the electrode charging. Soon after that, it begins to decrease because of the charge carrier's accumulation and restricted transport of injected charge carriers. Pondering to the charge carrier impeding behavior of the HTM/perovskite interface in the 3D cells, we believe that the drop of the SHG intensity is due to the accumulation of the charges at the HTM/perovskite interface. The time-dependent EFISHG measurements indicated that the local electric field adjusted in the perovskite film in the time range from 10^–6^ s to 10^–2^ s is due to the charges' injection and their transportation. These effects are different from the 2D/3D hybrid samples, where the HTM/perovskite interface has an interfacial layer. This interfacial 2D layer prevents the accumulation of charges at the HTM/perovskite interface. Further, the charge accumulation has been observed in both fresh and aged 3D samples (see Fig. 3b); however, no accumulation has been seen in 2D/3D hybrid cells (even in aged ones).

Furthermore, a close look at Fig. 3 indicates that the EFISHG signal intensity changed primarily in two stages. The initial increase of the EFISHG signals is due to the electrostatic field's development because of the charging of the Au electrode. In this stage, the externally applied field changes with time as E_e_ (t) = (*V*_ex_/*d*) (1 − exp(− *t*/*t*_RC_)) where *d* is the effective thickness of dielectric layers, and *t*_RC_ (which equals *R* × *C*) is the time constant of the RC circuit; where *R* is the resistance provided by the external circuit and *C* is the electrostatic capacitance of the PSCs. In the 2nd stage, a drop of the EFISHG wave intensity was observed (in the case of 3D), the M-W effect charge carriers stored at HTM/perovskite interfaces, and a space charge field (E_s_) is established, which leads to a decrease of SH intensity, and this phase occurs with a time response of *t*_MW_ known as (M-W charging). However, in the case of fresh 2D/3D hybrid samples, these two steps are obvious (Fig. 3c). The 2nd step in this graph probably demonstrates a better charge transfer in the fresh 2D/3D hybrid cells at the HTM/perovskite interface. However, in the case of the aged 2D/3D samples, it is difficult to distinguish these steps. It is important to note that the EFSIHG intensity is directly related to induced polarization. The higher the polarization, the higher the EFSIHG intensity, and the decrease in EFSIHG intensity with time can be directly linked to the degradation of this interface.

**Figure 4:**
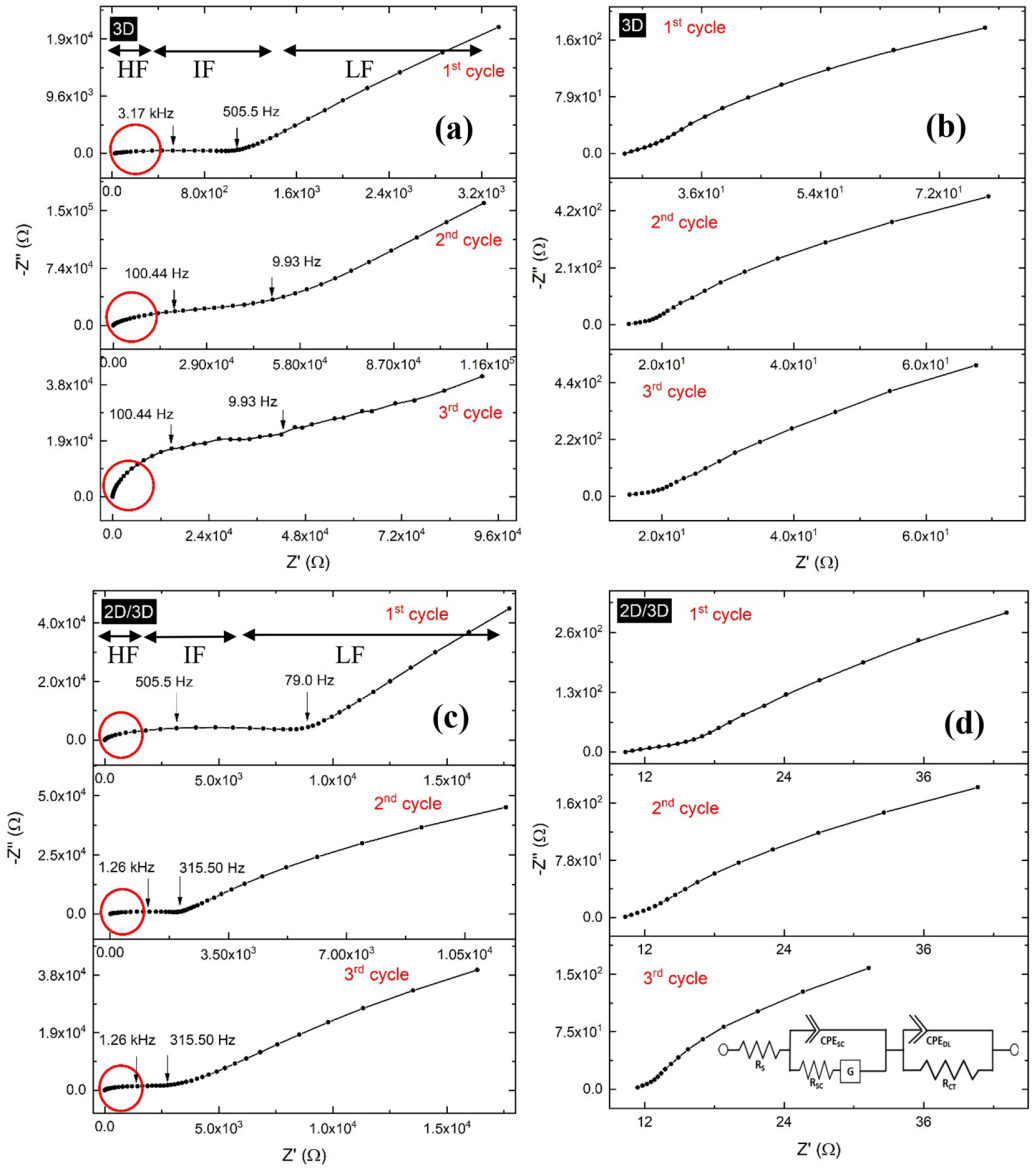
Impedance spectra of **(a)** the 3D and **(c)** 2D/3D hybrid PSCs recorded in three different cycles during the 5000 h with the time difference of 2500 h. **(b,d)** represent the HF region of the corresponding spectrum. These spectra were recorded under dark at 10 mV AC perturbation and 0 V DC bias.

Summarizing the above discussion, EFISHG data suggest that the Au/HTM/perovskite interface has progressively degraded with aging. In general, this indicates that the degradation and efficiency fading activities are strongly associated with the kinetics at the Au/HTM/perovskite interface, which we will further verify below. In the following sections, we demonstrate a detailed examination of methodical results obtained from the impedance spectroscopy (IS) characterization in order to get a further deep understanding of the underlying degradation processes from the viewpoint of a.c. signal response based on the general equivalent RC circuit model (see inset Fig. 4d).

To get a further insight into the Au/HTM/perovskite interface, impedance spectroscopy (IS) analysis of the 3D and 2D/3D PSCs was performed (under dark) over the aging period of 5000 h. Our device was modelled by using the equivalent RC circuit model to analyze the IS data, where R & C represent the resistive and capacitive components, respectively. The complex plane IS plots were gathered at the beginning, middle, and end of 5000 h. High-frequency (HF), intermediate-frequency (IF), and low-frequency (LF) notations were used to refer to the semicircles in the ranges of (1 MHz–1 kHz), (1 kHz–100 Hz), and (< 100 Hz), respectively. The HF arcs are referred to the formation of the space charge layer due to the electrode charging, while the IF arcs in the Nyquist plot correspond to charge transfer resistance (R_ct_) at the injection interface (Au/HTM/perovskite), whereas the LF arcs represent the recombination resistance. The capacitive component of the semicircle at HF corresponds to the geometrical capacitance^6,14^, while the parallel resistance component represents resistance provided by the space charge layer due to the electrode charging. In principle, the time constant associated with the HF and IF arcs should remain unchanged if the Au/HTM/perovskite interface is stable. However, the HF arcs shrank in both cases to 16 ± 2 kΩ cm^2^ within 2500 h, and remained constant afterward. The corresponding admittances increased by ~ 1.5 orders of magnitude (see fitting parameters in Table 3). Hence, the shrunk HF arcs suggest a decrease in the Au/HTM interface-resistances of both 3D and 2D/3D hybrid cells. The decrease in the resistance can be assumed by increasing the electric contact area caused by the densification or/and penetration of the Au into the HTM interface with time. In the HF region, the high value of the admittance (in the range of 10^–6^ S s^1/2^ cm^2^) in 2D/3D samples may be due to the smooth geometry of the Au/HTM interface. The results reported by Cho et al.^19^ using the SEM analysis showed that the 2D perovskite (over the 3D) coating leads to a smoother morphology as compared to the 3D based PSCs. During the 1st 2500 h, the IF arc in the 3D samples seems to increase in size intruding in the LF domain while, on the other hand, the intermediate arc is found to decrease in the 2D/3D hybrid cells encroaching towards the HF region as shown in Figs. 4b, d. The IF arc peak in the 3D samples shifted from the higher frequency of 3.17 kHz to the lower frequency of 100.44 Hz, while in the case of 2D/3D samples, it moved in the opposite direction (from 505.5 Hz to 1.26 kHz). No further shift has been observed after 2500 h. The resistance of the IF arc increased from 1.19 ± 0.3 Kω cm^2^ to 75.5 ± 6.7 kΩ cm^2^ in 3D cells, while in the case of 2D/3D hybrid cells, it decreased from 9.95 ± 1.2 kΩ cm^2^ to 2.8 ± 0.75 kΩ cm^2^ as shown in Table 3. The total impedance of the 3D cells increased by a factor of ~ 63.5 while it decreased in 2D/3D samples by a factor of ~ 3.5, within 5000 h.

**Table 3:** The fitting parameters extracted using the EEC given in Fig. 4d.

Sample	Cycle	Rs	HF region (electrode charging)			IF region (charger transfer)		
		(kΩ cm^2^)	R	Qy (S s^1/2^ cm^2^)	k _(_s^−1^_)_	R_ct_	Qy (S s^−1/2^ cm^2^)	k _(_s^−1^_)_
			(Ω cm^2^)			(kΩ cm^2^)	(10^–9^)	
3D	1st cycle	24.1 ± 0.3	35 ± 2	29.3 ± 3.1 (10^–9^)	0.824 ± 0.04	1.19 ± 0.3	181.25 ± 5.8	0.877 ± 0.06
	2nd cycle	14.2 ± 0.7	16 ± 1	37.7 ± 3.6 (10^–9^)	0.916 ± 0.02	73.2 ± 5.1	206.39 ± 6.1	0.721 ± 0.03
	3rd cycle	13.7 ± 0.6	16 ± 1	39.7 ± 4.2 (10^–9^)	0.958 ± 0.03	75.5 ± 6.7	307.43 ± 4.6	0.731 ± 0.05
2D/3D	1st cycle	12.0 ± 0.3	23 ± 2	28 ± 2.1 (10^–6^)	0.609 ± 0.05	9.95 ± 1.2	98.57.6 ± 4.2	0.912 ± 0.01
	2nd cycle	10.0 ± 0.2	17 ± 1	36 ± 2.5 (10^–6^)	0.588 ± 0.06	3.1 ± 0.85	119.39 ± 5.1	0.910 ± 0.02
	3rd cycle	9.0 ± 0.2	16 ± 1	44 ± 3.3 (10^–6^)	0.557 ± 0.04	2.8 ± 0.75	127.56 ± 5.4	0.899 ± 0.02

HF and IF stand for the high-frequency and intermediate-frequency, respectively.

A closer look at the data shows that; initially, the value of R_ct_ of 3D samples is much lesser than that of 2D/3D samples, which indicates that the quality of the HTM/perovskite junction was very good initially; but it degraded with the aging time. The spectrum of the 3D cells also looks disturbed after 5000 h. Even though the HTM layer acts as a protective layer to some extent, oxygen and moisture can still penetrate due to pinholes in the HTM layer^28^. Moreover, the doped HTM layer itself is known to degrade over time due to the presence of Lithium and Cobalt ions^29^. An accumulation of positively charged ions (cations) and negatively charged ions (anions) on either side of the perovskite absorber layer serve as recombination centers at the interface of transport layers that hinder the transport of charge carriers. Notably, the accumulation of the migrated ions at the transport layer interfaces leads to increased recombination centers, thereby increasing the transport resistance. This effect is evident in the 3D cells (as indicated in the EFISHG results), but the minima and maxima of the 2D layer lie in-between those of the 3D perovskite and HTM layers (as shown in Fig. 1). This provides an additional step for charge transfer and hence makes it easier for the charge carriers to travel across the interfaces instead of accumulation. The 2D layer protects the 3D perovskite layer from environmental conditions, and hence it stays stable for more extended periods^2^, as can be observed in Fig. 4, where the charge transfer resistance of the 2D/3D sample is much lower than that of the 3D sample exposed for the same aging period. The swapping of the values of the Z′ and Z″ peak frequencies at IF oppositely suggests distinctive degradation mechanisms at the HTM/perovskite interface in the 3D and 2D/3D cells.

The R_ct_ trend in the case of the 3D sample can be explained by the morphology of 3D perovskite with the passage of time. During the aging period, bigger grains of 3D perovskite provide enough intermolecular room to HTM grains; hence HTM layer collapses across the 3D perovskite layer and makes Au/HTM/perovskite interface more irregular. On the other hand, in the case of the 2D/3D PSC, a layer of 2D perovskite sandwiched between the 3D perovskite layer and the HTM film provides better inter-molecular contact between HTM and perovskite layer, which stays stable for an extended period of time, resulting in better charge transport across Au/HTM/perovskite; and the penetration of the Au into the HTM may significantly reduce the R_ct_ value. The shift of the R_ct_ toward the high frequency in 2D/3D hybrid cells is also due to the Au penetration in the HTM layer, and the charge transfer directly from the Au → 2D → 3D by diminishing the role of HTM to the perovskite layer. Hence, by minimizing the role of HTM, the charge extraction is supposed to decrease, and this results in efficiency fading. However, the opposite frequency response of R_ct_ in the 3D cells reflects the degradation of the Au/HTM/perovskite interface due to the collapse of the HTM layer across the 3D perovskite, and the charge transfer may take place directly from the Au → 3D layer.

Furthermore, the electrical equivalent circuit (EEC) fitting of all spectra's HF loop cannot be done using simple R||C or R||Q elements. The diffusion component was very significant; and to model the HF arc we used the Gerischer diffusion element (G) in series with "R". We found that the HF diffusion can be best fitted with G instead of the Warburg diffusion element (W), even though we used both and selected G due to the restricted or finite diffusion because of the formation of the space charge layer at the Au/HTM interface. It is important to mention that the Gerischer element consists of two factors: "complex admittance (unit S s^1/2^)" and a constant rate factor (k) (unit: s^−1^). Even though at HF, the Gerischer diffusion seems identical to the Warburg diffusion, but the Gerischer element represents the homogeneous diffusion while the Warburg element is best representing semi-infinite/unrestricted linear diffusion on a large planar electrode. However, at the Au/HTM interface, the Schottky junction's formation at the metal/semiconductor interface restricts the diffusion that can be best represented by G.

Recapping the above IS results, we realize that the impedance and the frequency response of the HF arc change quantitatively with aging. The PSCs' impedance data indicates a strong dependence of frequency on aging, which is demonstrated very clearly in the complex plane plots and can be presented using a parameterized model of the frequency-dependent electrical response using EEC. The characteristic frequency values reduce with time, and all area-specific admittance decrease with time, as well. The reducing admittance values indicate decreased or reduced HF and IF related interfaces in both types of cells. The impedance of the 2D/3D hybrid cells in the MF region decreases with aging, confirming that the interfacial resistance or the charge transfer resistance of the Au/HTM/perovskite interface decreases. Penetration of Au into the HTM, degradation of the perovskite layer, and charge accumulation altogether may affect the efficiency fading in the 3D samples, while in the 2D/3D hybrid samples, the impact of the degradation of the Au/HTM interface is more visible.

## Conclusion

In this paper, the degradation of 2D and 2D/3D hybrid PSCs were studied after aging tests conducted throughout 5000 h. The charge accumulation and change in charge transfer resistance were observed by I–V, IS, and EFISGH characterization methods, which illustrate the changes at the Au/HTM/perovskite interfaces of both types of the PSCs. Although the charge dynamics at the Au/HTM/perovskite interface in the 3D and 2D/3D hybrid cells behave oppositely with aging, they both reflect the occurrence of uncontrolled changes. Electrochemically induced reactions at the Au/HTM/perovskite interface happen possibly in both of 3D and 2D/3D hybrid cells and result in the degradation of this interface. This reveals that the integral structural instability of the perovskite layer may improve by using the 2D layer; however, degradation processes cannot be suppressed entirely with the 2D layer.

By evaluating the EFISHG and IS data, it may be postulated that the accumulation of charge significantly adds to the IF region's interfacial process in the 3D cells. The charge accumulation largely impedes the charge mobility at the HTM/perovskite interface, leading to higher charge transfer resistance in the 3D samples. The HTM/perovskite interface in 2D/3D hybrid cells shows relatively less degradation with aging, indicating that the 2D layer can minimize the decomposition of perovskite caused by environmental factors. Even though the thin 2D protecting layer's use enhanced the PSCs' performance and stability, this is not a complete solution to make the PSCs last for long operational life.

This work offers a further understanding of PSCs' underlying degradation processes, which are entrenched with the disintegration of the Au/HTM/perovskite interface. The findings also revealed critical future directions for the cathode interface engineering to alleviate the damages at the Au/HTM/perovskite interface. Future experiments are needed to concentrate on engineering the stable Au/HTM/perovskite interface with a higher stability window. Our understanding of the mechanistic analysis is yet at the initial phase, and more experiments are needed to attain a further comprehensive depiction.

## Data Availability

The data that support the findings of this study are available from the corresponding author upon reasonable request.
